# Heat-related illness among workers in British Columbia, Canada: Extreme hot weather in 2021 compared to 2001–2020

**DOI:** 10.5271/sjweh.4179

**Published:** 2024-10-01

**Authors:** Xiaocong Guo, Kate R Weinberger, Lillian Tamburic, Cheryl E Peters, Christopher B McLeod

**Affiliations:** 1 The University of British Columbia, School of Population and Public Health, Vancouver, British Columbia, Canada.; 2 ICF, Reston, Virginia, United States.; 3 BC Centre for Disease Control, Population and Public Health, Vancouver, British Columbia, Canada.

**Keywords:** case-crossover study, climate change, extreme heat, global warming, heat-related illness, heatwave, occupational health, workers’ compensation claim

## Abstract

**Objectives:**

British Columbia (BC), Canada, experienced an unprecedented summer with record-breaking high temperatures in 2021. Yet the health impact has not been examined in occupational settings. This study aimed to characterize occupational heat-related illness (HRI) among BC workers estimated by incidence rates and associations between heatwaves and HRI, compare risks from 2021 and prior summers of 2001–2020, and assess differential impacts on worker groups by demographics and occupations.

**Methods:**

We identified HRI from workers’ compensation claims that occurred between June and August from 2001–2021 in BC. Incidence rates were calculated using working population estimates from Statistics Canada’s Labour Force Survey. A time-stratified case-crossover design with conditional Poisson regression was used to examine the impact of heatwaves on occupational HRI. All analyses were stratified by year (2021 versus 2001–2020), age, sex, and occupation.

**Results:**

Of the 521 claims identified, 107 (21%) occurred in 2021. Incidence rates for 2021 and prior summers were 3.97 [95% confidence interval (CI) 3.26–4.80] and 0.93 (95% CI 0.85–1.03) claims per 100 000 workers, respectively. This difference represents a 327% increase. Rates were higher in health occupations in 2021 versus 2001–2020. During 2001–2021, the risk of HRI during heatwave days was 4.33 (95% CI 2.98–6.27) times that during non-heatwave days, and the risk was higher among middle-aged workers and workers in trades, transport, and equipment operations. The 2021 heatwaves had greater impact on younger and female workers than those from prior summers.

**Conclusions:**

Heat is a crucial workplace hazard. Prevention strategies should prioritize at-risk workers and not be limited to heatwaves.

In the summer of 2021, record-breaking high temperatures occurred in the Pacific Northwest region of Canada and the United States (US). Many locations experienced prolonged and intense extreme hot weather, including British Columbia (BC) in Canada, and Washington and Oregon in the US (1). In BC, the reported daily maximum temperature was 49.6° C in Lytton, and broke the Canadian national temperature record by 4.6° C, which stood for 86 years. It also exceeded the previous record among regions north of 45° N latitude set in July 1936 (1).

Heatwaves (ie, extreme heat events) are periods of abnormally hot weather. The 2021 heatwaves caused detrimental health impacts in the general population. The 2021 heatwave that occurred in late June contributed to 619, 100, and 83 heat-related deaths in BC (2), Washington (3), and Oregon (4), respectively. The risk of deaths in the general population during the heatwave was significantly higher among older populations and females (5). The health impacts were not limited to deaths but also include non-fatal outcomes. Increased rates of heat-related emergency department visits were observed in Washington and Oregon states, with higher risk among older populations and males (6, 7).

Workers are a group in a unique situation during periods of higher outdoor and indoor temperatures due to their limited ability to control heat exposure while at work. Some unique work-related factors may influence the thermal comfort of workers, such as radiant heat, physical exertion, protective clothing, and personal protective equipment (8). In addition, the working population and the general population vary in their demographic composition and general health conditions. Accordingly, the magnitude and distribution of health impacts of sustained extreme heat exposure in occupational settings cannot be generalized from the findings derived from the general population (9).

High temperatures can place workers at higher risk for heat-related illness (HRI), which is an acute effect ranging from mild heat edema to life-threatening heat stroke. The risk of HRI for workers is significantly higher than for the general population (9, 10). However, the majority of evidence is from regions with hot climates (11, 12). In BC, where most people reside and work in areas with a temperate climate, only one study characterized occupational HRI and found higher rates of lost-time claims for HRI among male workers, younger workers, and those working in primary industry. However, it examined neither the impact of heatwaves nor the effect of extreme heat experienced in 2021 (13). The sparse evidence from the working population in temperate climates limits our ability to understand how heatwaves impact workers in these climates. Knowledge specific to this climate can help in the further refinement of appropriate prevention strategies.

To generate this foundational knowledge, we examined workers’ compensation claims to (i) characterize occupational HRI among BC workers and compare risks from the unprecedented 2021 and prior summers of 2001–2020, with risks being estimated by incidence rates and associations between heatwaves and HRI; and (ii) assess how HRI incidence rates and heatwave impacts vary across age, sex, and occupational groups. This can help prioritize at-risk workers for the development and improvement of prevention strategies to mitigate risks for HRI in the workplace in BC’s changing climate.

## Methods

### Study area

Data was collected in BC, a Canadian province on the Pacific coast, at latitudes between 48° N and 60° N. Generally, the climate in BC is temperate throughout the year. Southern coastal BC has a mild climate moderated by the North Pacific current. In contrast, southern interior valleys have hot and dry summers influenced by continental air masses, with greater temperature extremes. The central and northern interior regions of BC account for two-thirds of the province’s land mass, but are largely mountainous and sparsely populated, with cool summers (14).

### Case identification

We identified occupational HRI cases from workers’ compensation claims from 2001 to 2021, provided by WorkSafeBC, which is the provincial workers’ compensation system and covers 92.5–98.2% of the labour force during the study period (15). WorkSafeBC investigates whether the injury or illness was caused by work or the work environment to determine if a claim is accepted, and reports the occurrence date of the injury or illness based on information provided by the worker and the employer. Sufficient work exposure must be established in order for a claim to be accepted. In the case where non-work exposure may have contributed to the injury or illness, the work exposure must be shown to have contributed to the injury or illness on the balance of probabilities (ie, the work exposure was more likely than not to have caused the injury or illness). Claim adjudicators make this determination in consultation with medical advisors. Either the worker or employer has the right to appeal the claim eligibility decision for internal review and, following this, to an external appeal tribunal. Only accepted workers’ compensation claims for HRI that resulted in time lost from work were included in our analyses. The less severe claims that resulted in health care utilization but no time lost from work were not included because the compensation system lacked information about the cause of such claims.

We identified HRI claims based on International Classification of Diseases, ninth revision or nature of injury or source of injury codes. The diagnostic codes are presented in table 1. We further restricted our cohort to workers aged ≥15 years and those with no missing information reported on relevant covariates (ie, age, sex, occupation, employer location, meteorological data). We limited the analysis to summer months from 1 June to 31 August to minimize the impact of seasonality.

**Table 1:** Diagnostic codes for identification of lost-time claims for heat-related illness

Diagnosis scheme & code		Description
International Classification of Diseases, 9^th^ revision		
	992.0	Heat stroke and sun stroke
	992.1	Heat syncope
	992.2	Heat cramps
	992.3	Heat exhaustion, anhidrotic
	992.4	Heat exhaustion, due to salt depletion
	992.5	Heat exhaustion, unspecified
	992.6	Heat fatigue, transient
	992.7	Heat edema
	992.8	Other unspecified heat effects
	992.9	Unspecified effects of heat and light
	276.5	Dehydration
Nature of Injury		
	00240	Heat stroke, sun stroke, heat exhaustion
	07200	Effects of heat and light, unspecified
	07210	Heat stroke
	07220	Heat syncope
	07230	Heat fatigue
	07240	Heat edema
	07280	Multiple effects of heat and light
	07290	Effects of heat and light, not elsewhere classified
Source of Injury		
	93620	Heat-environmental

### Meteorological data

Historical meteorological data were obtained from Daymet (16), a daily weather dataset providing gridded estimates on a 1×1 km surface resolution. The gridded daily estimates from inhabited land were further spatially averaged across forward sortation areas (FSA), which is a geographic area based on the first three digits of Canadian postal codes and inhabited land was selected based on Statistics Canada’s population ecumene boundary (ie, a population density of ≥0.4 residents per square kilometer) (17). This can ensure more accurate exposure measures and minimize impact by large areas of forest or bodies of water.

In this study, daily maximum temperature and mean water vapor pressure were retrieved, and the daily mean vapor pressure was used to calculate the daily mean relative humidity using Bolton’s equation (18), as relative humidity is likely to be a confounder of the heatwave-HRI relationship by influencing evaporative heat loss (11, 19). The FSA-level daily meteorological data were linked to HRI claims based on the date of occurrence of HRI and employer location reported on claims, assuming constant daily exposure among all workers within each FSA.

Heatwaves were defined as two or more consecutive days with summertime daily maximum temperature above the 95^th^ percentile of temperatures for each FSA over all summer days between 2001 and 2020, as used in previous studies (20–23). Such location-based relative thresholds can better capture the spatial variation in workers’ acclimatization to the regional climate.

### Statistical analyses

A descriptive analysis was conducted to characterize HRI claims that occurred over 2001–2021 and by age group, sex, and occupation. We further compared the HRI claims from the 2021 summer to those from the 2001–2020 summers. The occupation categories were defined based on the highest level of the National Occupation Classification hierarchy (24).

Incidence rates were calculated as the number of HRI claims per 100 000 BC workers, where the denominator was the count of BC workers estimated from the Labor Force Survey (25). We calculated the annual average rates for the summertime period, the average rates of the 2021 summer compared to that of prior summers from 2001–2020, and by age, sex, and occupation. The 95% confidence intervals (CI) were calculated using Byar’s approximation of the exact Poisson distribution (26).

To examine the impact of heatwave days on HRI claims, a time-stratified case-crossover study design was adopted, such that each case day was matched to 3–4 referent days based on the day of the week, month, and year. As comparisons are made within individuals, this study design eliminates confounding by known and unknown factors that do not vary day-to-day (27). We modeled daily counts of HRI with heatwaves using conditional Poisson regression. Relative humidity was adjusted for in the multivariable regression models as a time-varying confounder. The analyses were stratified by year (ie, 2021 versus 2001–2020), age, sex, and occupation. All analyses were conducted in R version 4.0.5 (28).

## Results

There were 557 lost-time HRI claims that occurred between 1 June and 31 August during 2001–2021, of which 521 had complete data and were included in the analysis. The distribution of HRI is characterized in table 2. The claims that occurred in 2021 (N=107) represents 21% of the total number of claims over the entire 21-year study period. Compared to prior summers, the HRI claims in 2021 were more likely to occur among workers aged ≥55 years, female workers, and those with occupations in health and business, finance, and administration. There were fewer HRI claimants in 2021 who were aged 15–24, male, and working in processing, manufacturing, and utilities.

**Table 2:** Summary statistics and incidence rates of accepted lost-time claims for heat-related illness among workers in British Columbia, Canada, with claims that occurred between 1 June and 31 August, 2001–2021: Statistics compared between 2021 summer and 2001–2020 summers. [CI=confidence interval]

Variable		2001–2021			2001–2020			2021	
		Claims N (%)	Rate (95% CI) per 100 000 workers per year		Claims N (%)	Rate (95% CI) per 100 000 workers per year		Claims N (%)	Rate (95% CI) per 100 000 workers per year
Overall		521 (100)	1.11 (1.02–1.21)		414 (79.5)	0.93 (0.85–1.03)		107 (20.5)	3.97 (3.26–4.80)
Age group (years)									
	15–24	94 (18.0)	1.31 (1.06–1.60)		80 (19.3)	1.18 (0.93–1.47)		14 (13.1)	3.59 (1.96–6.03)
	25–34	147 (28.2)	1.48 (1.25–1.73)		114 (27.5)	1.22 (1.00–1.46)		33 (30.8)	5.48 (3.77–7.69)
	35–44	102 (19.6)	0.97 (0.79–1.18)		83 (20.0)	0.83 (0.66–1.03)		19 (17.8)	3.26 (1.96–5.09)
	45–54	111 (21.3)	1.03 (0.85–1.24)		88 (21.3)	0.86 (0.69–1.06)		23 (21.5)	4.35 (2.76–6.53)
	≥55	67 (12.9)	0.78 (0.61–0.99)		49 (11.8)	0.62 (0.46–0.81)		18 (16.8)	3.06 (1.81–4.83)
Sex									
	Male	362 (69.5)	1.46 (1.31–1.62)		299 (72.2)	1.28 (1.14–1.43)		63 (58.9)	4.42 (3.40–5.65)
	Female	159 (30.5)	0.72 (0.61–0.84)		115 (27.8)	0.55 (0.45–0.66)		44 (41.1)	3.47 (2.52–4.66)
Occupation									
	Primary industry	61 (11.7)	4.17 (3.19–5.35)		48 (11.6)	3.43 (2.53–4.55)		13 (12.1)	20.14 (10.71–34.44)
	Trades, transport, and equipment operators and related	251 (48.2)	3.45 (3.03–3.90)		209 (40.1)	3.02 (2.63–3.46)		42 (39.3)	11.38 (8.20–15.38)
	Processing, manufacturing, and utilities	41 (7.9)	2.31 (1.65–3.13)		36 (8.7)	2.12 (1.48–2.94)		5 (4.7)	6.23 (2.01–14.54)
	Art, culture, recreation, and sport	16 (3.1)	0.98 (0.56–1.59)		– ^a^	0.78 (0.40–1.36)		– ^a^	4.43 (1.19–11.34)
	Sales and service	81 (15.5)	0.65 (0.52–0.81)		63 (15.2)	0.54 (0.41–0.69)		18 (16.8)	2.81 (1.67–4.45)
	Natural and applied sciences and related	16 (3.1)	0.49 (0.28–0.79)		– ^a^	0.43 (0.23–0.73)		– ^a^	1.18 (0.24–3.44)
	Social science, education, government service, and religion	13 (2.5)	0.33 (0.18–0.56)		– ^a^	0.30 (0.15–0.54)		– ^a^	0.67 (0.07–2.40)
	Health	20 (3.8)	0.64 (0.39–0.98)		8 (1.9)	0.27 (0.12–0.54)		12 (11.2)	5.51 (2.85–9.63)
	Business, finance, and administration	19 (3.6)	0.24 (0.15–0.38)		11 (2.7)	0.15 (0.07–0.26)		8 (7.5)	1.81 (0.78–3.58)

^a^ Statistics are not presented for occupation categories with <5 claims in 2021.

**Figure 1:**
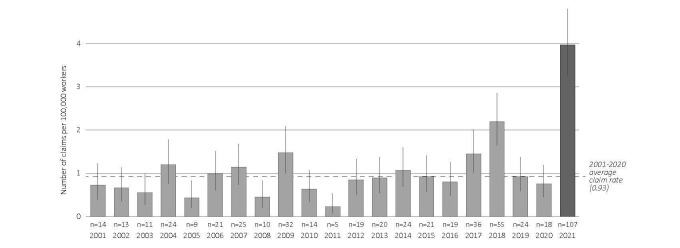
Annual incidence rates for the summertime period of accepted lost-time claims for heat-related illness per 100 000 workers in British Columbia, Canada, 2001–2021. The dotted line indicates the overall average incidence rate for the 2001–2020 summers.

The 521 HRI claims corresponded to a rate of 1.11 (95% CI 1.02–1.21) per 100 000 workers per summer. As presented in figure 1, there was no visually apparent temporal trend in prior summers, with rates for individual years ranging from 0.23 (95% CI 0.07–0.53) claims per 100 000 in 2011 to 2.19 (95% CI 1.65–2.86) per 100 000 in 2018. The claims from 2021 (N=107) corresponded to the highest rate for any year of 3.97 (95% CI 3.26–4.80) per 100 000. The difference between summer 2021 and the prior summer average (0.93, 95% CI 0.85–1.03) represented a 327% increase (table 2).

The characteristics of HRI incidence rates by age, sex, and occupation are summarized in table 2. Over 2001–2021, we found higher rates of HRI among workers who were younger, male, and in occupations from primary industry; trades, transport, and equipment operators; and processing, manufacturing, and utilities. Rates in 2021 were intensified compared to prior averages. The greatest increases were observed among workers in the 25–34 and 45–54 age groups and in occupations from primary industry; trades, transport, and equipment operators; and health occupations.

**Figure 2:**
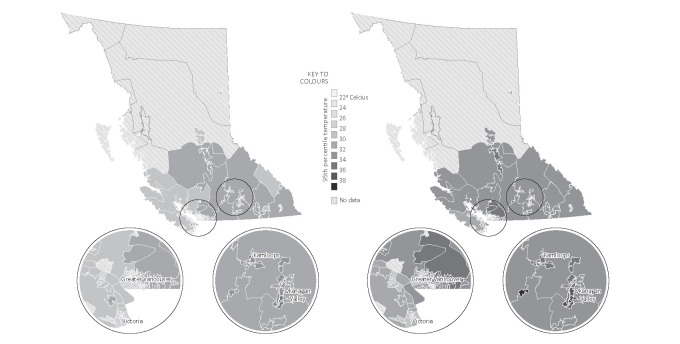
The distribution of 95^th^ percentiles of summertime daily maximum temperatures across forward sortation areas in British Columbia, Canada, in prior summers of 2001–2020 (left) versus summer 2021 (right).

The daily ambient temperatures in summer 2021 were more extreme compared to prior summers. The 95^th^ percentiles of summertime daily maximum temperatures for FSA ranged from 21.0 to 34.9 in prior summers, and from 22.2 to 38.6 in summer 2021 (figure 2). The annual average number of heatwave days per FSA was two in prior summers versus seven in summer 2021. Of the 521 HRI claims, 135 (26%) occurred during heatwave days. The associations between heatwaves and HRI are reported in table 3, and associations stratified by occupation are only reported for the two occupational groups with the highest claim counts. The overall risk of HRI during heatwave days was 4.33 (95% CI 2.98–6.27) times that during non-heatwave days, when controlling for relative humidity. The relative risk increased from 3.24 (95% CI 2.11–4.98) in prior summers to 10.19 (95% CI 4.45–23.35) in summer 2021. Across the entire 21-year study period, the impacts of heatwaves were greater among workers aged 45–54, 25–34, and 15–24, as well as workers from trades, transport, and equipment operators. The risk of HRI did not differ significantly between heatwave and non-heatwave days among workers aged ≥55 and in primary industry. The 2021 heatwaves had greater impact in HRI among the younger age groups of 25–34 and 15–24 and in female workers, when compared to prior summers.

**Table 3:** Associations between heatwaves and heat-related illness among workers in British Columbia, Canada, between 1 June and 31 August from 2001–2021: Statistics compared between 2021 summer and 2001–2020 summers. [RR=relative risk; CI=confidence interval]

Variable		2001–2021 N=521		2001–2020 N=414		2021 N=107
		RR (95% CI)		RR (95% CI)		RR (95% CI)
Overall		4.33 (2.98–6.27) ^a^		3.24 (2.11–4.98) ^a^		10.19 (4.45–23.35) ^a^
Age group (years)						
	15–24	5.42 (2.35–12.46) ^a^		3.84 (1.51–9.77) ^a^		20.18 (2.33–174.50) ^a^
	25–34	6.54 (3.04–14.05) ^a^		3.99 (1.69–9.43) ^a^		43.63 (4.57–416.71) ^a^
	35–44	2.23 (1.00–4.96) ^a^		1.82 (0.73–4.53)		2.78 (0.38–20.29)
	45–54	12.77 (3.72–43.80) ^a^		10.67 (2.33–48.84) ^a^		27.23 (2.61–284.15) ^a^
	≥55	1.77 (0.64–4.93)		1.83 (0.54–6.25)		0.77 (0.08–7.10)
Sex						
	Male	4.41 (2.80–6.95) ^a^		3.69 (2.21–6.15) ^a^		8.33 (2.88–24.11) ^a^
	Female	4.33 (2.23–8.43) ^a^		2.48 (1.11–5.68) ^a^		13.53 (3.54–51.62) ^a^
Occupation ^b^						
	Primary industry	2.01 (0.56–7.20)		1.70 (0.36–8.12)		3.50 (0.44–28.02)
	Trades, transport, and equipment operators and related	3.36 (2.00–5.63) ^a^		2.96 (1.63–5.35) ^a^		4.83 (1.55–15.08) ^a^

^a^ Statistically significant with P<0.05. ^b^ Relative risks by occupation were only reported for the two occupational groups with the highest claim counts.

## Discussion

The incidence rate of HRI claims was 327% higher during the extremely hot summer of 2021 compared to prior summers among workers aged ≥15 years in BC. The risk of HRI during heatwave days was significantly higher than that during non-heatwave days, and this association was greater in magnitude during summer of 2021 in contrast to prior summers. In 2021, there was a notable increase in rates from health occupations, while heatwaves of this year had greater association with HRI among younger and female workers than those from prior summers. Over the entire 21-year study period, heatwaves had a greater impact in elevating risks of HRI among workers aged 45–54 years and those working in trades, transport, and equipment operators. Incidence rates were higher among workers who were younger, male, and in occupations with substantial manual and outdoor work.

The risk of HRI in workplaces during heatwave days was 4.33 times that during non-heatwave days. This impact may have been greater absent any protective measures, such as WorkSafeBC’s advisory to cease work during extreme heat events (29). Our finding was similar as observed in South Australia, which found 4–7 times the risk in a city located at latitude of 35° S (12). This is more than 10° closer in latitude to the equator compared to BC. These findings indicate elevated risk regardless of latitudes and regional climates, although the heatwave thresholds vary due to varying sensitivity to heat in those climates.

The annual average incidence rate for HRI in BC was 1.11 per 100 000 workers, which is similar to that of other Canadian provinces of Ontario (1.62 per 100 000) (30) and Quebec (1.04 per 100 000) (11), but considerably lower than that of South Australia (4.5 per 100 000) (12), and states of Florida (3.5 per 100 000) (9) and Washington (3.1 per 100 000) (31) in the US. Yet comparison of incidence rates across regions warrants cautious interpretation because of different administrative datasets and study periods selected to identify occupational HRI. In addition, the incidence rate is likely an underestimate of the true burden of occupational HRI due to underreporting of HRI claims. Reasons may include that workers may think HRI is a mild health issue and not worth the time and effort to file a claim, they have job security and future employment concerns, or they may lack knowledge about the eligibility and coverage of workers’ compensation (32).

In summer 2021, the distribution of HRI and the impact of more intense and prolonged extreme heat presented different trends as compared to prior summers. Incidence rates were much higher, and heatwaves had a greater association with HRI. Compared to prior summers, heatwaves in 2021 were attributed to a higher risk of HRI among younger workers in age groups 15–24 and 25–34, as well as female workers. It is possible that younger workers were more likely to assume they are healthier and chose not to take preventive actions and were more likely to undertake more strenuous work (33). The impact of heatwaves on HRI became greater among female workers than male workers, which contrasts with prior summers. This reversal indicates that female workers may be less heat tolerant than male workers at extremely elevated temperatures (34). In addition, health professionals experienced a more noticeable increase in the incidence rates. The findings comparing 2021 versus prior summers should be interpreted with caution as several factors may modify the anomalous trend observed in 2021. One factor possibly contributing to this finding is the fact that some workers (eg, health professionals, essential workers working indoors) had to wear additional personal protective equipment during the COVID-19 pandemic, which may impede heat dissipation and increase their risk of HRI (35). Another factor modifying the trend may be behavior change during the COVID-19 pandemic. For example, among workers who were able to work from home, the risk of HRI may be modified by the availability of air conditioning (AC) at home. In BC, only 32% households had AC, of which most were found in interior regions with hot summers and in those with higher socioeconomic status (36). A possible third factor is the lack of acclimatization as the most intense and prolonged heatwave in 2021 occurred in the early summer, when workers were poorly adjusted to the sudden increase in ambient temperatures. This is particularly important for workers undertaking strenuous work and those who are not acclimatized to wearing masks in hot environments. This aligns with US studies which found that occupational HRI cases were more likely to occur during the first week of employment (31) and in early summer (23) when workers were not physiologically adapted to tolerate heat and not psychologically vigilant to take preventative actions.

Middle-aged workers experienced greater impact from heatwaves and younger workers were at higher risk of HRI over summer months. The impact of heatwaves was higher among the 45–54 age group, suggesting that middle-aged workers may have deteriorated thermoregulatory functions and limited tolerance to extreme heat (37). Although older workers are expected to experience more physiological strain with advancing age (37), we did not observe significant differences in the risk of HRI during heatwave and non-heatwave days among workers aged ≥55 years. We hypothesize that these workers were more likely to work in more senior roles in offices equipped with AC or other cooling interventions. Healthy worker effect may also play a role in that workers who are more susceptible to heat may self-select to depart from their positions, leaving the fittest workers in the jobs (38). In contrast, incidence rates were highest among younger workers during the summer months. This aligns with a previous study which found that the proportion of claimants <25 years old who filed HRI claims was significantly greater than that of all compensation claims (31). This is possibly because young workers were more likely to be assigned physically demanding tasks or entry-level labor positions (13, 30). Prevention strategies should be developed considering variations in heat susceptibility among age groups in response to varying degrees of heat exposure.

BC workers had 4.33 times the risk of HRI during heatwave compared to non-heatwave days, and the risks were not significantly different between men (4.41) and women (4.33), which was similarly observed in a South Australian study (11). In contrast, the overall incidence rates of HRI were higher among male versus female workers. These findings reflect gender differences as reported in previous studies (11, 12, 31), which could be explained by the fact that male workers constitute approximately 80% of the workforce that engages in more strenuous and/or outdoor work (39). When restricting to specific occupations, female workers had similar or higher rates compared to male workers (13). In combination with our unique findings from the comparison of 2021 to prior average, prevention strategies should not be limited to male workers, and should be dynamic, taking into consideration the level of heat intolerance, especially during prolonged exposure to extreme heat.

The risk of HRI was disproportionate by occupation. The three occupations with the highest incidence rates were those in primary industry (eg, agriculture, forestry); trades, transport, and equipment operators (eg, construction, transportation); and processing, manufacturing, and utilities (eg, machine operators). The findings were similar to those observed in Washington State (US) in that HRI rates were 5.2, 12.1, 3.5, and 3.0 per 100 000 workers for agriculture, forestry, fishing and hunting; construction; transportation; and manufacturing, respectively (31). Although workers from primary industry had the highest incidence rate, their risks during heatwave days were not significantly different from non-heatwave days. We hypothesize that these workers were more likely to perform high-exertion activity at work. The increased metabolic rate induced by strenuous activity may contribute to the increased internal body heat, which may place them at higher risk for HRI even when the temperatures were not extreme (40). These workers were also more likely to work outdoors with radiant heat from the sun and lack of effective cooling interventions, which may contribute to the increased risk of HRI (41). Future research may examine the relationships between ambient temperatures and HRI to determine the temperature thresholds at which risks are elevated in different subgroups. Prevention strategies should target high-risk occupations for a wide range of weather conditions, not only limited to heatwave days.

Our study has some limitations. First, the incidence rates may not reflect the true burden of occupational heat exposure in BC because the lost-time claims did not capture HRI cases that were not reported to WorkSafeBC and those that did not result in time lost from work. More research is warranted to capture HRI cases more comprehensively, including those that were not severe enough to result in time lost from work and those that were work-related but underreported to workers’ compensation systems. Identifying more cases may allow for more granular stratified analysis by combinations of age, sex, and/or occupation to account for disproportionate demographics within occupations to identify at-risk workers and allow better estimates of the true HRI incidence. In addition, future research may further explore the impact of heatwaves on workplace injury, which has been well documented in other jurisdictions (42). Second, there may be exposure misclassification particularly in sectors where workers predominantly work in remote sites, different from their reported employer’s locations (eg, forestry workers). Such non-differential misclassification may bias the results towards the null. In addition, using FSA-level meteorological estimates to represent heat exposure for all workers within each FSA may decrease precision, but is not expected to introduce bias in effect estimates (43). However, they cannot capture the full variability of microenvironments at workplaces (eg, presence of cooling or indoor heat sources). Ideally, to more precisely characterize heat exposure, future research should consider collecting information on work-specific heat sources or objectively measuring heat in workplaces, particularly in indoor work environments. Third, we did not consider relative humidity, solar radiation, and/or wind speed in heat metrics to define heatwaves as doing so is to estimate the combined effect of ambient temperature and additional weather parameters on HRI. Future studies may adopt additional heat metrics such as humidex and wet bulb globe temperature to better reflect workers’ experience of heat exposure and provide additional insight with respect to differential susceptibilities to heat.

This study has several strengths. It is the first study to quantify the association between occupational HRI and heatwaves in BC using workers’ compensation data linked with meteorological data. The near-universal coverage of workers’ compensation data allows the findings to be generalized to all BC workers aged ≥15 years. Another strength is the use of time-stratified case-crossover study design which accounts for time-invariant confounding and minimizes potential bias arising from time trends. In addition, our study is the first to compare results from the 2021 summer, which experienced record-breaking extreme temperatures, to prior summers in occupational settings. This provides insight about varying levels of heat susceptibilities to heatwaves of extreme intensity and informs prevention and adaptation strategies to mitigate the effects of intense future heatwaves in this warming climate. A further strength of our study is that we not only examined risk factors for incidence of occupational HRI during summer months, but also assessed whether those at-risk subgroups were at higher risk during heatwave compared to non-heatwave days. This points to HRI being not only a result of prolonged and intense heat exposure and informs broader prevention measures even in the absence of heatwaves. Future work should explore the association between ambient temperatures and occupational HRI and identify risk factors for HRI when temperatures are high but not extreme. Further, heatwaves were defined by using location specific thresholds. This allows generalizability of our results to regions in different climates.

### Concluding remarks

Occupational heat exposure is a crucial workplace hazard that contributes to increased risk of HRI among workers, even in regions that typically feature temperate climates. The risk of HRI will likely increase due to the trend of warming temperatures and extremes in weather. Our findings of different at-risk subgroups during heatwaves can inform the development and improvement of prevention strategies to mitigate occupational HRI for these workers. Prevention strategies should prioritize at-risk subgroups in response to not only heatwaves but also high ambient temperatures to improve the health and safety of workers in BC.

### Ethics approval

Ethical approval for this study was obtained from the Behavioural Research Ethics Board of the University of British Columbia (H22-01396).

### Disclaimer

All inferences, opinions, and conclusions drawn in this study are those of the authors and do not reflect the opinions or policies of the Data Stewards.

## Data Availability

The gridded daily weather estimates on a 1×1 km surface resolution can be obtained via Daymet (https://daac.ornl.gov/cgi-bin/dsviewer.pl?ds_id=2129). Workers’ compensation data used in this study were obtained via data access agreement with the data steward and are not publicly available.

## References

[1] White RH, Anderson S, Booth JF, Braich G, Draeger C, Fei C et al. The unprecedented Pacific Northwest heatwave of June 2021. Nat Commun 2023 Feb;14(1):727. 10.1038/s41467-023-36289-3 36759624 PMC9910268

[2] BCCS. Extreme heat and human mortality: A review of heat-related deaths in B.C. in summer 2021 [Internet]. British Columbia: British Columbia Coroners Service; 2022 Jun [cited 2023 Nov 6]. Available from: https://www2.gov.bc.ca/assets/gov/birth-adoption-death-marriage-and-divorce/deaths/coroners-service/death-review-panel/extreme_heat_death_review_panel_report.pdf

[3] Washington State Department of Health. Heat Wave 2021 [Internet]. [cited 2023 Nov 6]. Available from: https://doh.wa.gov/emergencies/be-prepared-be-safe/severe-weather-and-natural-disasters/hot-weather-safety/heat-wave-2021

[4] Oregon Office of Emergency Management. State of Oregon initial after-action review (AAR) of the June 2021 excessive heat event [Internet]. Oregon Office of Emergency Management; 2021 Jul [cited 2023 Nov 6]. Available from: https://digital.osl.state.or.us/islandora/object/osl:973559

[5] Henderson SB, McLean KE, Lee MJ, Kosatsky T. Analysis of community deaths during the catastrophic 2021 heat dome: early evidence to inform the public health response during subsequent events in greater Vancouver, Canada. Environ Epidemiol 2022 Jan;6(1):e189. 10.1097/EE9.0000000000000189 35169667 PMC8835552

[6] Schramm PJ, Vaidyanathan A, Radhakrishnan L, Gates A, Hartnett K, Breysse P. Heat-Related Emergency Department Visits During the Northwestern Heat Wave - United States, June 2021. MMWR Morb Mortal Wkly Rep 2021 Jul;70(29):1020–1. 10.15585/mmwr.mm7029e1 34292925 PMC8297695

[7] Nori-Sarma A, Milando C, Weinberger KR, Hess JJ, Errett NA, Wellenius GA. Association Between the 2021 Heat Wave in Portland, Oregon, and Seattle, Washington, and Emergency Department Visits. JAMA 2022 Dec;328(23):2360–2. 10.1001/jama.2022.20665 36538316 PMC9856788

[8] WorkSafeBC. Preventing Heat Stress at Work. WorkSafeBC; 2023 Jul. Report No.: BK30.

[9] Florida Department of Health. Assessing the relationship of ambient temperature and heat related illness in Florida: implications for setting heat advisories and warnings. Florida Department of Health, Division of Disease Control and Health Protection, Bureau of Epidemiology; 2012.

[10] Fortune M, Mustard C, Brown P. The use of Bayesian inference to inform the surveillance of temperature-related occupational morbidity in Ontario, Canada, 2004-2010. Environ Res 2014 Jul;132:449–56. 10.1016/j.envres.2014.04.022 24866772

[11] Adam-Poupart A, Smargiassi A, Busque MA, Duguay P, Fournier M, Zayed J et al. Summer outdoor temperature and occupational heat-related illnesses in Quebec (Canada). Environ Res 2014 Oct;134:339–44. 10.1016/j.envres.2014.07.018 25199975

[12] Xiang J, Hansen A, Pisaniello D, Bi P. Extreme heat and occupational heat illnesses in South Australia, 2001-2010. Occup Environ Med 2015 Aug;72(8):580–6. 10.1136/oemed-2014-102706 26081622

[13] Weinberger KR, Tamburic L, Peters CE, McLeod CB. Heat-Related Illness Among Workers in British Columbia, 2001-2020. J Occup Environ Med 2023 Feb;65(2):e88–92. 10.1097/JOM.0000000000002761 36730139

[14] Geography Open Textbook Collective. British Columbia in a global context [Internet]. British Columbia: BCcampus Open Education; 2014 [cited 2023 Nov 14]. Available from: https://opentextbc.ca/geography/chapter/9-2-introduction/

[15] AWCBC/ACATC. Detailed Key Statistical Measures Report [Internet]. 2023 [cited 2023 Oct 24]. Available from: https://awcbc.org/en/statistics/ksm-annual-report/

[16] Thornton MM, Shrestha R, Wei Y, Thornton PE, Kao SC, Wilson BE. Daymet: Daily Surface Weather Data on a 1-km Grid for North America, Version 4 R1 [Internet]. Oak Ridge, Tennessee, USA: ORNL DAAC; 2022 [cited 2023 Oct 25]. Available from: https://daac.ornl.gov/cgi-bin/dsviewer.pl?ds_id=2129

[17] Statistics Canada. Population Ecumene Census Division Cartographic Boundary File, Reference Guide, 2016 Census [Internet]. Statistics Canada; 2017 Feb [cited 2023 Oct 25]. Report No.: Catalogue no. 92-159-G. Available from: https://www150.statcan.gc.ca/n1/pub/92-159-g/92-159-g2016001-eng.htm#:~:text=2.-,Overview,have%20made%20their%20permanent%20home

[18] Williams PD, Ambaum MH, editors. Chapter 5 - Water in the atmosphere. In: Thermal Physics of the Atmosphere [Internet]. Second Edition. Elsevier; 2021 [cited 2023 Oct 25]. p. 91–114. (Developments in Weather and Climate Science). Available from: https://www.sciencedirect.com/science/article/pii/B9780128244982000124

[19] Parsons K. Human Thermal Environments: The Effects of Hot, Moderate, and Cold Environments on Human Health, Comfort, and Performance [Internet]. Third Edition. Boca Raton: CRC Press; 2003 [cited 2024 Feb 12]. 635 p. Available from: https://www.taylorfrancis.com/pdfviewer/

[20] Smith TT, Zaitchik BF, Gohlke JM. Heat waves in the United States: definitions, patterns and trends. Clim Change 2013 Jun;118(3-4):811–25. 10.1007/s10584-012-0659-2 23869115 PMC3711804

[21] Tian Z, Li S, Zhang J, Guo Y. The Characteristic of Heat Wave Effects on Coronary Heart Disease Mortality in Beijing, China: A Time Series Study. Barengo NC, editor. PLoS ONE. 2013 Sep 30;8(9):e77321. 10.1371/journal.pone.0077321 PMC3786924 24098818

[22] Kanti FS, Alari A, Chaix B, Benmarhnia T. Comparison of various heat waves definitions and the burden of heat-related mortality in France: implications for existing early warning systems. Environ Res 2022 Dec;215(Pt 2):114359. 10.1016/j.envres.2022.114359 36152888

[23] Shire J, Vaidyanathan A, Lackovic M, Bunn T. Association Between Work-Related Hyperthermia Emergency Department Visits and Ambient Heat in Five Southeastern States, 2010-2012-A Case-Crossover Study. GeoHealth. 2020;4(8):e2019GH000241. 10.1029/2019GH000241 PMC7429406 32821873

[24] Government of Canada. Hierarchy and structure [Internet]. 2023 [cited 2024 Feb 19]. Available from: https://noc.esdc.gc.ca/Structure/Hierarchy

[25] Statistics Canada. Labour Force Survey (LFS) [Internet]. 2023 [cited 2023 Nov 23]. Available from: https://www.statcan.gc.ca/en/survey/household/3701

[26] Rothman K, Boice J. Epidemiologic Analysis with a Programmable Calculator. Washington DC: National Institutes of Health; (NIH Publication 79-1649).

[27] Sun S, Weinberger KR, Nori-Sarma A, Spangler KR, Sun Y, Dominici F et al. Ambient heat and risks of emergency department visits among adults in the United States: time stratified case crossover study. BMJ 2021 Nov;375:e065653. 10.1136/bmj-2021-065653 34819309 PMC9397126

[28] R Core Team. R: A language and environment for statistical computing [Internet]. Vienna, Austria: R Foundation for Statistical Computing; 2022. Available from: https://www.R-project.org/

[29] WorkSafeBC. WorkSafeBC advising employers to consider workplace closures during heat wave [Internet]. 2021 [cited 2023 Nov 6]. Available from: https://www.worksafebc.com/en/about-us/news-events/news-releases/2021/June/worksafebc-advising-employers-to-consider-workplace-closures-during-heat-wave

[30] Fortune MK, Mustard CA, Etches JJ, Chambers AG. Work-attributed illness arising from excess heat exposure in Ontario, 2004-2010. Can J Public Health 2013 Sep;104(5):e420–6. 10.17269/cjph.104.3984 24183186 PMC6973883

[31] Bonauto D, Anderson R, Rauser E, Burke B. Occupational heat illness in Washington State, 1995-2005. Am J Ind Med 2007 Dec;50(12):940–50. 10.1002/ajim.20517 17972253

[32] Safe Work Australia. Work-related injuries in Australia, 2005-2006: Factors affecting applications for workers’ compensation [Internet]. 2009 [cited 2023 Nov 13]. Report No.: ISBN 978–0–642–32890–8. Available from: https://www.safeworkaustralia.gov.au/system/files/documents/1702/work_related_injuries_2005_06_factors_affecting_application_wc.pdf

[33] Centers for Disease Control and Prevention. Preventing deaths, injuries, and illnesses of young workers [Internet]. 2003 [cited 2023 Nov 14]. Report No.: DHHS (NIOSH) Publication No. 2003-128. Available from: https://www.cdc.gov/niosh/docs/2003-128/pdfs/2003128.pdf

[34] Kazman JB, Purvis DL, Heled Y, Lisman P, Atias D, Van Arsdale S et al. Women and exertional heat illness: identification of gender specific risk factors. US Army Med Dep J 2015;(9617037):58–66. 26101907

[35] Davey SL, Lee BJ, Robbins T, Randeva H, Thake CD. Heat stress and PPE during COVID-19: impact on healthcare workers’ performance, safety and well-being in NHS settings. J Hosp Infect. 2021;108(id6, 8007166):185–8. 10.1016/j.jhin.2020.11.027 PMC7720696 33301841

[36] Quick M, Tjepkema M. The prevalence of household air conditioning in Canada [Internet]. Statistics Canada; 2023 Jul [cited 2023 Nov 16]. Report No.: 10.25318/82-003-x202300700002-eng. Available from: https://www150.statcan.gc.ca/n1/pub/82-003-x/2023007/article/00002-eng.htm 37470464

[37] Blatteis CM. Age-dependent changes in temperature regulation - a mini review. Gerontology 2012;58(4):289–95. 10.1159/000333148 22085834

[38] Xiang J, Bi P, Pisaniello D, Hansen A. Health impacts of workplace heat exposure: an epidemiological review. Ind Health 2014;52(2):91–101. 10.2486/indhealth.2012-0145 24366537 PMC4202759

[39] Statistics Canada SC. Statistics Canada. 2023 [cited 2023 Nov 15]. Proportion of women and men employed in occupations, annual, inactive. Available from: https://www150.statcan.gc.ca/t1/tbl1/en/tv.action?pid=1410033502

[40] Mac V, Elon L, Mix J, Tovar-Aguilar A, Flocks J, Economos E et al. Risk Factors for Reaching Core Body Temperature Thresholds in Florida Agricultural Workers. J Occup Environ Med 2021 May;63(5):395–402. 10.1097/JOM.0000000000002150 33560064

[41] Xiang J, Bi P, Pisaniello D, Hansen A. The impact of heatwaves on workers’ health and safety in Adelaide, South Australia. Environ Res 2014 Aug;133:90–5. 10.1016/j.envres.2014.04.042 24906072

[42] Varghese BM, Hansen A, Nitschke M, Nairn J, Hanson-Easey S, Bi P et al. Heatwave and work-related injuries and illnesses in Adelaide, Australia: a case-crossover analysis using the Excess Heat Factor (EHF) as a universal heatwave index. Int Arch Occup Environ Health 2019 Feb;92(2):263–72. 10.1007/s00420-018-1376-6 30406332

[43] Weinberger KR, Spangler KR, Zanobetti A, Schwartz JD, Wellenius GA. Comparison of temperature-mortality associations estimated with different exposure metrics. Environ Epidemiol 2019 Oct;3(5):e072. 10.1097/EE9.0000000000000072 33195965 PMC7608890

